# A Tale of 4 Valves: Migration of Transcatheter Mitral Valve Requiring Surgical Intervention

**DOI:** 10.1016/j.atssr.2023.02.014

**Published:** 2023-03-02

**Authors:** Motahar Hosseini, Mackram F. Eleid, Juan A. Crestanello

**Affiliations:** 1 Department of Cardiovascular Surgery, Mayo Clinic, Rochester, Minnesota; 2 Department of Cardiovascular Medicine, Mayo Clinic, Rochester, Minnesota

## Abstract

Severe mitral annular calcification increases the risk associated with conventional mitral valve replacement. We report a case of surgical mitral valve replacement after migration of 3 transcatheter mitral valves in a patient with severe mitral annular calcification.

Mitral valve replacement (MVR) in the setting of mitral annular calcification (MAC) is a challenging surgical procedure that is associated with an increased incidence of complications. Transseptal transcatheter MVR (TMVR) in the setting of MAC has become an alternative option with its own shortcomings.^1^

The patient is a 60-year-old woman with history of restrictive pulmonary disease, morbid obesity, and rheumatoid arthritis receiving immunosuppressive therapy. She presented with New York Heart Association class III heart failure symptoms secondary to severe mitral valve (MV) stenosis (MV diastolic mean gradient, 20 mm Hg; area, 0.88 cm^2^) and severe pulmonary hypertension (mean pulmonary artery pressure, 58 mm Hg; pulmonary capillary wedge pressure, 34 mm Hg; pulmonary vascular resistance, 5.53 Wood units; left atrial pressure, 33 mm Hg; left ventricular [LV] systolic pressure, 106 mm Hg; LV end-diastolic pressure, 21 mm Hg on invasive hemodynamic catheterization).

She was deemed high risk for conventional MVR because of her comorbidities and severe MAC. Therefore, she was referred for valve-in-MAC TMVR. Cardiac computed tomography showed severe, nearly circumferential MAC, with severely thickened leaflets and severe leaflet calcification especially of the posterior leaflet (MAC calcium score of 9).^2^ Mitral annular measurements (Figure 1) included trigone-trigone distance of 23.7 mm (diastolic) and 24.5 mm (systolic), D-shaped perimeter of 85.6 mm (diastolic) and 103 mm (systolic), and D-shaped annular area of 803 mm^2^ (diastolic) and 780 mm^2^ (systolic).

**Figure 1:**
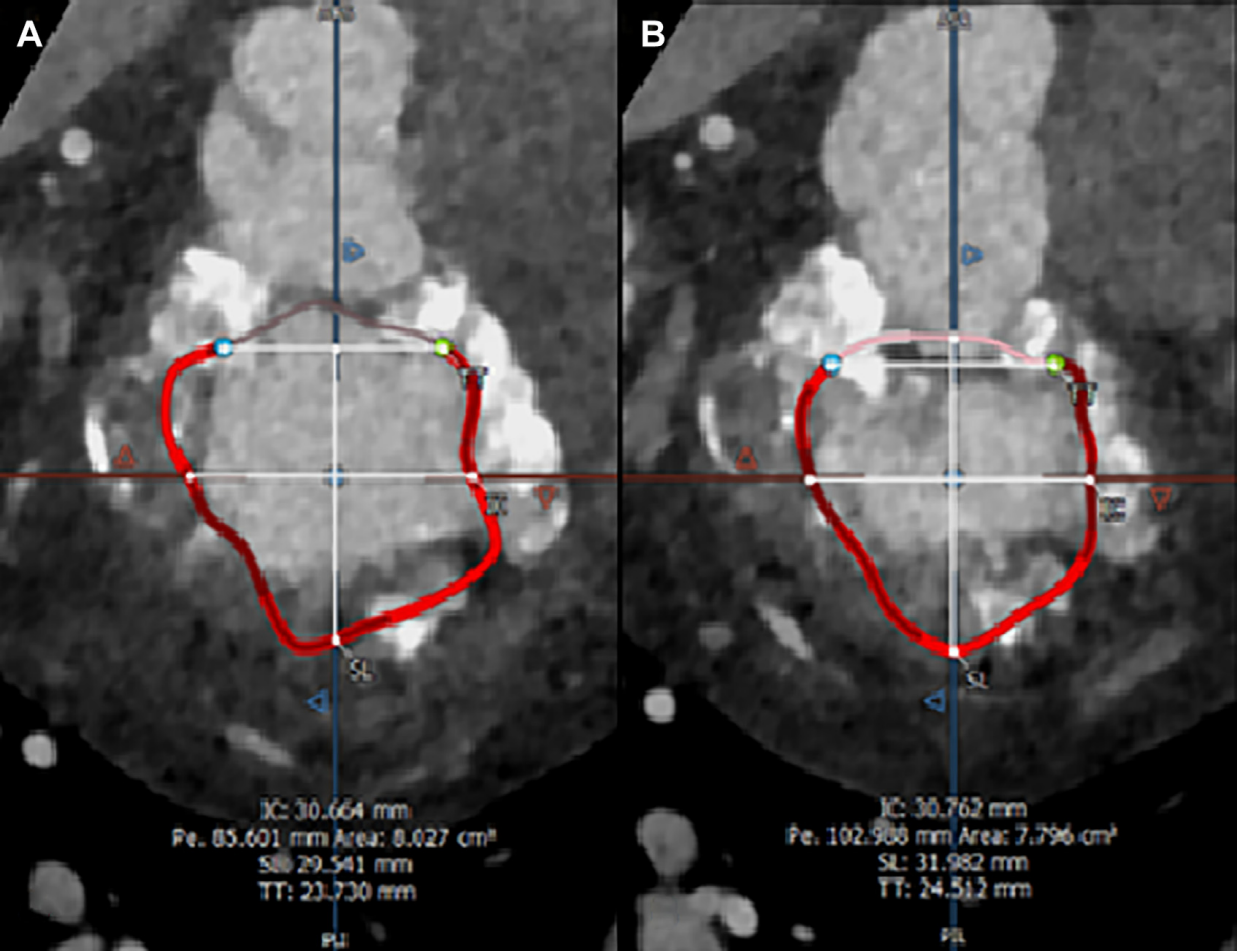
Mitral annular measurements. (A) Diastolic measurements: trigone-trigone distance, 23.7 mm; D-shaped perimeter, 85.6 mm; and D-shaped annular area, 803 mm^2^. (B) Systolic measurements: trigone-trigone distance, 24.5 mm; D-shaped perimeter, 103 mm; and D-shaped annular area, 780 mm^2^.

In preparation for TMVR, she underwent laceration of the anterior mitral leaflet (AML) to prevent outflow obstruction (LAMPOON) procedure to further eliminate the risk of neo–LV outflow tract obstruction.^3^ This was followed by transseptal valve-in-MAC TMVR as part of the Mitral Implantation of TRAnscatheter vaLves II (MITRAL-II) trial with 3 overlapping SAPIEN 3 valves (Edwards Lifesciences; NCT04408430; Figure 2).^4^ The first valve (26-mm SAPIEN 3 Ultra + 5 mL added volume) migrated atrially immediately after deployment, necessitating deployment of a second valve (26-mm SAPIEN 3 Ultra + 5 mL added volume) to anchor the first valve inside the annulus. There was severe paravalvular leak (PVL) as well as unstable motion of both valves with ongoing atrial migration. It was decided to proceed with deployment of a larger valve, 29-mm SAPIEN 3 valve (with 4 mL added volume), and subsequent balloon dilation (29-mm SAPIEN 3 valve balloon + 6 mL added volume). This improved the existing PVL. After the procedure, the patient remained in New York Heart Association class III. Transthoracic echocardiography 6 weeks after the procedure demonstrated a mean mitral gradient of 7 mm Hg and moderate to severe MV PVL. Repeated cardiac catheterization showed improvement in pulmonary hypertension with significant decrease in left-sided filling pressures (mean pulmonary artery pressure, 37 mm Hg; pulmonary capillary wedge pressure, 14 mm Hg; left atrial pressure, 13 mm Hg; LV systolic pressure, 110 mm Hg; LV end-diastolic pressure, 16 mm Hg) and pulmonary vascular resistance (3.66 Wood units). It was decided to proceed with surgery. The operation was performed through a median sternotomy. Cardiopulmonary bypass was instituted with aortic and bicaval cannulation. The left atrium was accessed through a transseptal approach, and the SAPIEN valves were explanted (Figure 3). The AML was split by LAMPOON, and it was protruding into the left atrium (not shown). The remaining AML was excised. No additional leaflet resection was performed. Circumferential sutures of 2-0 Ethibond were placed in the MV annulus with pledgets in the ventricular side. The valve was sized for a 31-mm bioprosthesis (Hancock II porcine bioprosthesis; Medtronic). The patient was weaned from cardiopulmonary bypass successfully. Dismissal transthoracic echocardiography showed a diastolic gradient across the mitral bioprosthesis of 5 mm Hg and no PVL. Postoperative recovery was uneventful.

**Figure 2:**
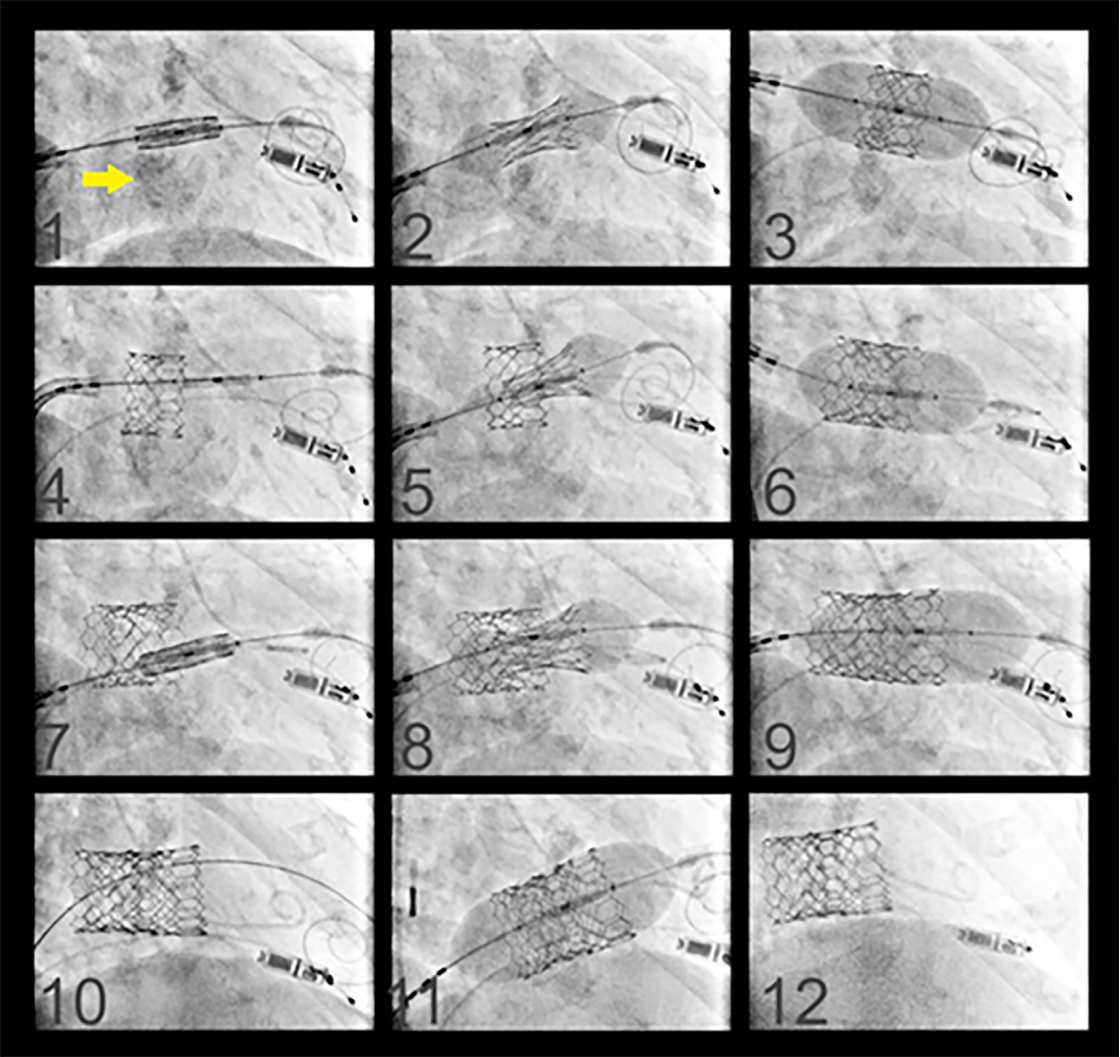
Step-by-step approach to transcatheter mitral valve replacement showing mitral annular calcification (arrow) and the valve in respect to the annulus.

**Figure 3:**
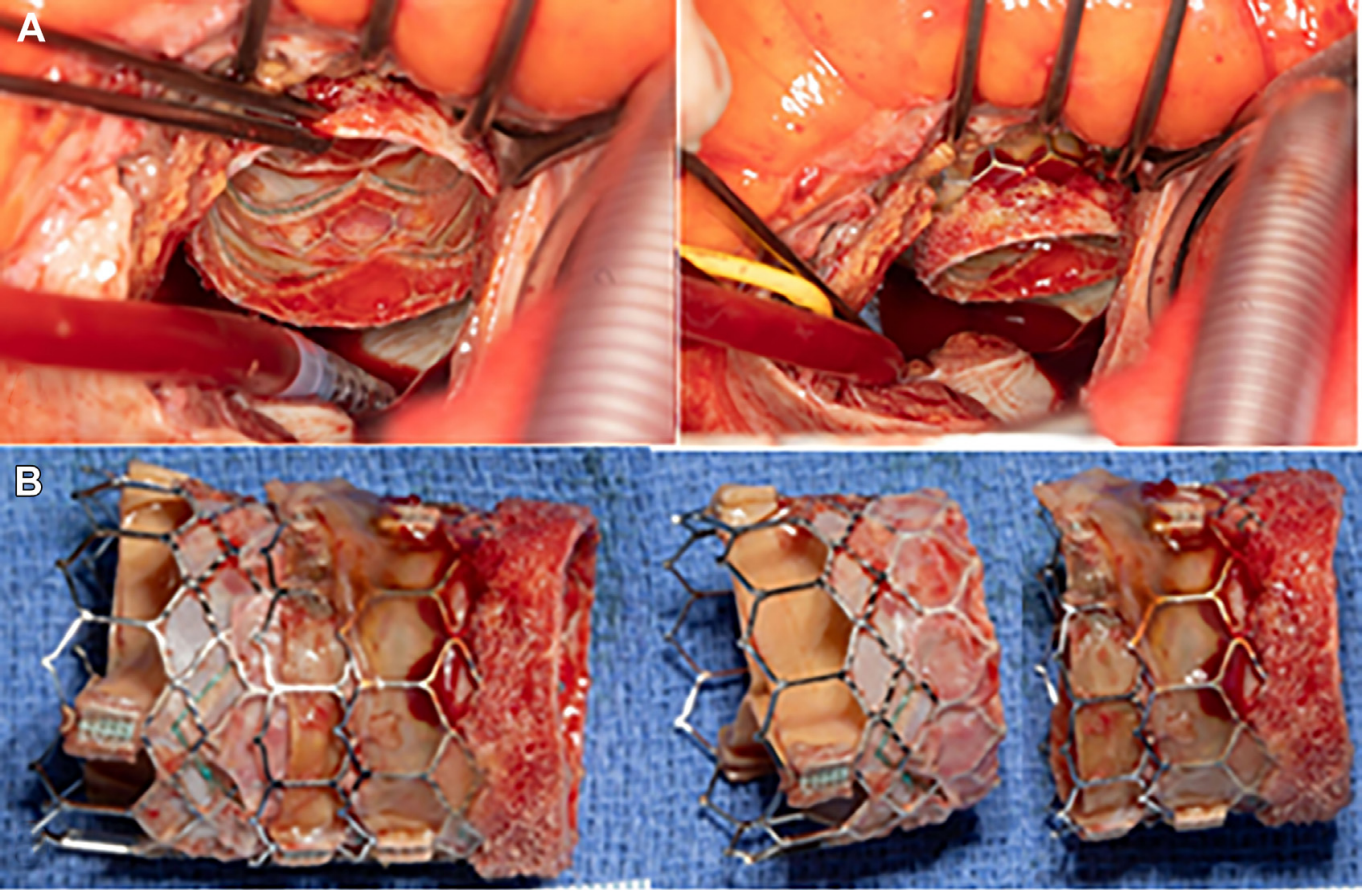
View of the transcatheter mitral valves from the left atrium (A) before explantation and (B) after explantation.

## Comment

MAC is a degenerative noninflammatory process resulting from the deposition of calcium along the mitral annulus.^5^ The calcification most commonly involves the posterior annulus but can also involve the entire annulus, the leaflets, and the myocardium.^5^ Severe MAC influences the decision-making process for the treatment of MV dysfunction. Prevalence of MAC in the adult population is reported to be 23%, which is the same as the reported prevalence of MAC in patients undergoing isolated MVR.^6^^,^^7^

Patients with MAC have increased comorbidities, such as diabetes, dialysis, and peripheral vascular disease.^7^ Other valve diseases and coronary artery disease are more common in patients with concomitant MAC and MV disease.^6^ MAC is associated with 1.3-fold higher mortality.^6^ Patients who have concomitant MAC and MV disease have even higher mortality rates compared with patients with MAC without MV disease.^6^ Patients with MAC who undergo MVR demonstrate significantly worse survival compared with those without MAC.^7^ However, this worse survival appears to be the result of increased comorbidities, and MAC has not been shown to be an independent risk factor for mortality.^7^ Despite these observations and reported overall higher mortality in patients with concomitant MAC and MV disease, those patients who underwent MV intervention demonstrated better overall survival than patients who did not undergo intervention and were managed medically.^8^ Therefore, MV intervention (surgery or transcatheter), despite the risk and technical challenges, will improve survival. Transseptal TMVR in MAC has become an alternative option for these patients.

There are currently no specific guidelines for valve-in-MAC TMVR in the United States except for short-term data from reported cases series.^1^ Risks associated with valve-in-MAC TMVR using balloon-expandable valves designed for aortic position include LV outflow tract obstruction, device embolization and migration, PVL, and death.^1^ It has been shown that the severity of annular calcification has an important role in anchoring the valve to prevent embolization and migration.^2^ Patients with mild to moderate MAC who undergo TMVR with a balloon-expandable aortic prosthesis have the highest risk of embolization compared with patients with severe MAC.^2^ Factors associated with lower risk of embolization include a calcium thickness of ≥5 mm, calcium distribution of ≥270 degrees of annular circumference, anterolateral trigone, and AML calcification on cardiac computed tomography.^2^ In our patient, several factors may have contributed to the TMVR migration: the annular calcification appears “caseous” in nature with reduced MAC density to anchor the device (Figure 1); a smaller valve (26 mm) was implanted first; and AML laceration may have led to reduced anchoring of the valve. TMVR in turn may have facilitated surgical MVR by crushing the calcified annulus, allowing easier suture placement. Despite elevated surgical risk, conventional MVR is feasible in achieving valve competency with no PVL even in the setting of severe MAC. Additional studies are required to further define the best candidates for valve-in-MAC TMVR.
